# Insights into the Research Trends on Bovine Colostrum: Beneficial Health Perspectives with Special Reference to Manufacturing of Functional Foods and Feed Supplements

**DOI:** 10.3390/nu14030659

**Published:** 2022-02-04

**Authors:** Rahul Mehra, Renu Garhwal, Karnam Sangwan, Raquel P. F. Guiné, Edite Teixeira Lemos, Harpal Singh Buttar, Pradeep Kumar Singh Visen, Naveen Kumar, Anuradha Bhardwaj, Harish Kumar

**Affiliations:** 1 Amity Institute of Biotechnology, Amity University Rajasthan, Jaipur 303002, India; rahulmehranov@gmail.com (R.M.); renugarhwal7@gmail.com (R.G.); ksangwan3@gmail.com (K.S.); nkumar2@jpr.amity.edu (N.K.); 2 CERNAS Research Centre, Polytechnic Institute of Viseu, 3504-510 Viseu, Portugal; etlemos@esav.ipv.pt; 3 Department of Pathology and Laboratory Medicine, Faculty of Medicine, University of Ottawa, Ottawa, ON K1H 8M5, Canada; hsbuttar@bell.net; 4 Nutra-V Inc., Toronto, ON M1K 3B8, Canada; p.visen@utoronto.ca; 5 ICAR-National Research Centre on Equines, Hisar 125001, India; anu15virgo@gmail.com

**Keywords:** bovine colostrum, nutritional and bioactive components, food and feed supplements, prophylaxis

## Abstract

Bovine colostrum (BC) is the initial mammary secretion after parturition, which is nature’s bountiful source consisting of nutritional and bioactive components present in a highly concentrated low-volume format. All mammalian newborns require colostrum to enhance physiological processes such as lifelong immunity, gastrointestinal development, and resistance to microbial infections. The genetic, environmental, and processing methods can all have an impact on the biochemical contents of BC and its supplements. BC and its derivatives have been intensively researched for their potential use in functional foods, medicines, and animal feed. Evidence from clinical studies suggests that BC products are well-tolerated, nontoxic, and safe for human ingestion. Functional foods, feed, and pharmaceutical formulations based on bovine colostrum are playing noteworthy roles in the development of innovative products for promoting health and the prevention of chronic illnesses. This systematic review sheds light on recent research on (a) the effects of processing techniques on BC components, (b) emerging techniques used in the isolation and identification of novel components, (c) BC-based functional foods for human consumption and animal feed supplements, and (d) the role of BC in current drug delivery, as well as future recommendations.

## 1. Introduction

Around the world, a research trend is emerging in the exploitation of bovine colostrum (BC) and its derivatives in the development of functional foods and pharmaceuticals for the prevention of gastrointestinal and respiratory illnesses [1]. The natural presence of nutritious and physiologically active components such as immunoglobulins, growth factors, hormones, and lactoferrins drives the increased interest in BC. Generically, BC is a yellowish–reddish viscous fluid secreted from mammary glands immediately after parturition that is predominantly composed of nutritious and bioactive components in a low-volume high-density format [1,2,3]. The prime constituents of BC were categorized into (a) immune factors, namely immunoglobins (IgG, IgA, and IgM), lactoferrin (LF), lysozyme, lactoperoxidase, microRNA, glycoconjugates, B and T lymphocytes, leukocytes, interleukins, and other proline-rich polypeptides; (b) growth factors, namely insulin growth factors (IGF-I and -II), epithelial growth factors, growth hormones, platelet-derived growth factors, fibroblast growth factor, and epidermal growth factor; and (c) nutritional components, namely fatty acids, conjugated linoleic acid, oligosaccharides (major, neutral, and acidic), amino acids, vitamins, and minerals [4,5,6]. Excluding lactose, the levels of bioactive and nutritional components were elevated immediately after parturition and subsequently began to drop after calving [7,8].

The proteins and peptides secreted into milk by the mammary glands are responsible for milk’s bioactivity. Opioid peptides are peptides with a high affinity for opioid receptors and exhibit pharmacologic activities similar to morphine. They are enzymatically encoded (in vitro) from human and bovine β-casein and have been demonstrated to be present in the central nervous system, GI tract, and immune systems of animals [9]. The β-casomorphins are found in raw human and cow milk [10]. The N-terminal sequence of all regular opioid peptides is the same (Try-Gly-Gly-Phe). The whey protein encompasses opioid-identical sequences, specifically α-lactalbumin for both human and bovine at fragment 50–53 and β-lactoglobulin for bovine at fragment 102–105 [11]. Further, these peptides are termed α-lactorphin and β-lactorphin. Jarmołowska et al. [12] reported that the concentration of β-casomorphins (BMC5 and BMC7) was found to be higher in the human colostrum as compared to human milk. Furthermore, the concentration of β-casomorphins in human colostrum was 5-fold higher (5.03 μg/mL) for BMC5 and 8-fold higher (3.10 μg/mL) for BCM7 as compared to mature human milk (4 months) by the values of BMC5 (0.58) and BCM7 (0.33), respectively. Similarly, Nguyen et al. [13] reported that the concentration (ng/g milk) of BMC5 in raw cow milk samples ranged from 0.40 to 0.64, and that of BCM7 ranged from 0.76 to 8.41.

Immunoglobulins (Igs) are antibodies present in both cow and human colostrum and milk, and they constitute the main immunological protein components of the acquired immune system. Different classes of Igs, namely IgG, IgA, IgE, IgM, and IgD, were categorized based on their size, amino acid concentration, charge, and biological functions. These protein fractions were found in the serum and other body fluids of animals having slow β-electrophoretic mobility [14]. However, in bovine colostrum, IgG (IgG_1_ and IgG_2_) is the most prevalent antibody, accounting for 80–85% of total Igs, whereas in human colostrum, IgA is the most prominent antibody class [14,15]. IgG is delivered to neonates by the colostrum or the placenta; furthermore, IgG is the only antibody class that penetrates the human placenta substantially. The neonatal Fc receptor expressed on syncytiotrophoblast cells mediates this crossing [16]. The dominant immunoglobulin (IgG) in ruminants is derived primarily from blood or serum, with translocation through mammary alveolar cells driven by an active receptor mechanism. During the prepartum dry phase, immunoglobulins accumulate in the mammary gland of mammals and are eventually released in the colostrum [17]. The difference in the concentration of immunoglobulins (IgG, IgA, and IgM) between bovine/human milk and colostrum and serum is tabulated in Table 1.

**Figure 1:**
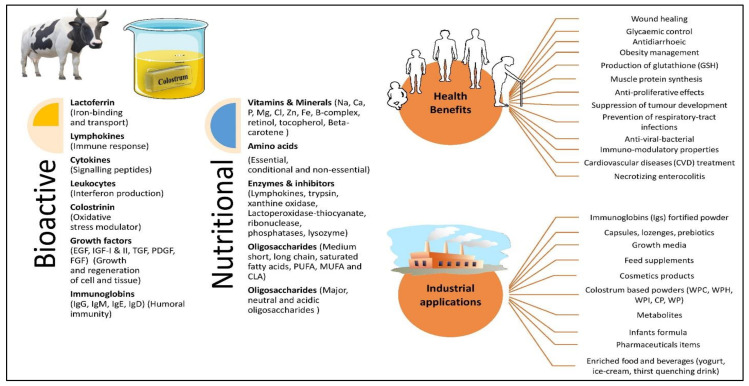
Overview of bovine colostrum nutritional and bioactive components with their medicinal and industrial applications.

The biochemical composition of BC is significantly altered by internal and external factors, including heat/cold stress, relative humidity (RH), elevation, breed, calving, age, dry period, seasons, and other genetic factors [7,8]. Furthermore, several authors reported that colostrum composition is considerably affected by the processing techniques and conditions, including irradiation, heating, dehydration, sonication, dehydration, filtration, and homogenization [13,18,19]. Aside from influencing composition, these strategies help to reduce microbial load, maintain consistency when added to commodities, and extend shelf life. Diverse cutting-edge technology and instruments including solid-phase extraction, transmission infrared (IR) spectroscopy, split trehalase immunoglobulin G assay (STIGA), proteomic analysis, isobaric tags for relative and absolute quantitation (iTRAQ), and lectin microarray profiling are effectively used by researchers for the isolation and identification of concealed colostrum constituents [20,21,22,23]. These technological innovations create tremendous possibilities for researchers and food manufacturers to develop a cost-effective, nontoxic, and health-promoting food/feed additive from bovine colostrum. The isolated, purified, or transformed constituents from BC are further exploited as a functional ingredient in the manufacture of a wide range of dietary supplements with many medicinal uses [24,25]. Recently, the approaches of exploring BC-based feed for feeding fish [26], broiler chickens [27], and birds [28] have been reported in the literature, and it has been demonstrated to be successful in delivering a suitable high protein content for overall body composition. Scientific evidence from placebo-controlled clinical trials indicates that BC components have a wide range of pharmacological uses in the prevention and pretreatment of acute and chronic diseases [29,30]. Figure 1 depicts an outline of the elements of bovine colostrum, as well as their health and industrial uses.

This review article intends to summarize the trend of bovine colostrum research, drawing from processing, innovations, role in food/feed, and prophylactic usage, based on the evidence obtained from scientific studies published in recent years.

## 2. Impact of Processing Techniques and Conditions on Colostrum Constituents

Food processing is intended to ensure the quality and safety of food while minimizing nutritional and bioactive constituent loss and maximizing deterioration of pathogenic and spoilage microorganisms with extended shelf life [31]. The industrial use of bovine colostrum on a large scale is limited due to its low coagulation temperature, microbial load, and other issues generated during processing and storage [32,33,34]. Several processing strategies, including pasteurization, microwave, spray drying, freezing, thermal treatments, drying techniques (freeze and spray drying), filtering, membrane processing, HPP, and pulse electric field, have been documented in the literature to reduce microbial burden in BC [35,36,37]. Traditional BC-based products such as kharvas, junnu, and posu (India); kalvdans (England); abrystir (Iceland); and råmelk (Norway) are popular in their respective nations. These puddings are often prepared by utilizing BC collected between the first and second days, followed by heating and the addition of mature milk together with sugar or spices. The thermal treatment applied to the BC mixture results in a gel-like structure. The process underlying gel formation following heating was investigated by Hege et al. [5]. While describing the findings, the authors mentioned that the gel-like structure of BC upon heating is associated with the presence and an elevated concentration of proteins, specifically β-lactoglobulin (β-La). Furthermore, during thermal treatment (65 to 71 °C for 30 min), these protein fractions in BC begin denaturing and rearranging themselves, eventually leading to the formation of gel-like structures formed by β-La in collaboration with other proteins such as lactoferrin, insulin growth factors I and II, N-acetylgalactosaminyltransferase-1, and minor proteins. These proteins contain a large percentage of cysteine, which establishes disulfide bonds with other proteins, leading to the permanent cross-links that contribute to the formation of a gel network. Moreover, the composition of colostrum, fat droplets, and ions has a substantial impact on the strength of the gel network. Additionally, the gelation temperatures (°C) of skim colostrum, diluted skim colostrum, skim colostrum UF retentate, colostrum whey, and colostrum whey UF retentate were 75.83 ± 0.17, 78.00 ± 0.29, 72.20 ± 0.17, 77.83 ± 0.017, and 74.50 ± 0.17, respectively [38].

Salar et al. [37] recommended that gentle pasteurization of BC (57 °C for 30 min) together with drying methods (freeze or spray drying) could act as hurdle technology which results in the reduction in microbial load with minimal impact on bioactive constituents (lactoferrin and IgG) and antioxidant activities of BC. Bovine colostrum immunoglobulin (IgG) was shown to be 15% denatured after long-time pasteurization (63 °C for 30 min) and 34% denatured after high-temperature, short-time pasteurization (72 °C for 15 s) by Chatterton et al. [39], while the IgG was completely inactivated by ultra-heat treatment [40]. Another study, conducted by Elizondo-Salazar et al. [41], indicates that the thermal treatment (60 °C or >60 °C for <1 h) completely denatured immunoglobulin IgG_1_ but had less effect on IgG_2_ concentration. Furthermore, the thermal treatment of BC at 60 °C for 30 min or 1 h significantly reduces bacterial load without affecting viscosity. Recently, Mann et al. [42] found that thermal treatment of colostrum at 63 °C for 60 min results in a reduction in viable bacteria (SCC and TPC), but simultaneously it also has a negative effect on the concentrations of growth factor (IGF-I) (10%), fibrinogen, insulin (22%), and IgA (8.5%) when compared to raw samples.

The impact of irradiation vs. heat treatment on the immunomodulatory characteristics of bovine colostrum was studied in a comparative study by Nguyen et al. [13]. The authors prepared four types of colostrum powder during the experiment: (a) without pasteurization, (b) pasteurized (63 °C for 30 min), (c) pasteurized (72 °C for 15 s), and (d) pasteurized powder followed by gamma radiation (14 kGy dose). The powders were tested against *Staphylococcus aureus*, *S. epidermidis*, and *Escherichia coli*, cytokine secretion and intestinal epithelial cell proliferation were assessed (in vitro). The study’s results indicate that heat treatments reduce the bioactive composition and the inhibitory and immunomodulatory capabilities of colostrum, while the heat treatment combined with gamma radiation assists in preserving sterility and minimizing bioactive component loss. The approach described above can be used in the manufacturing sector to archive products with low bacterial loads and minimal loss of bioactive components. Temperatures above 72 °C result in the loss of immunoglobulin (IgG) immune activities, which is associated with a conformational change in the secondary structure, whereas pulse electric fields (0–41.1 KV/cm; 0–91.4 us) do not cause perceptible changes in IgG functionality and secondary structure [43].

In the manufacture of dairy-based or non-dairy-based powders, both freezing and spray drying processes are universally acknowledged and frequently used to obtain products with minimum loss of bioactive components. In contrast, Wang et al. [44] found that both freezing and spray drying can be used to prepare lactoferrin (Lf) powder from bovine colostrum with minimal denaturation and conformation changes. Spray drying at 180 °C inlet and 95 °C outlet temperature was also recommended for the manufacturing of bovine lactoferrin powder, which has antioxidant activity nearly identical to raw bovine colostrum lactoferrin. Lactoferrin is lost throughout a temperature range of 65–95 °C as time and temperature increase [45]. Additionally, spray drying is efficient in suppressing the transmission of the bovine leukemia virus (BLV) in milk and colostrum to some extent [46].

High-pressure processing (HPP) is a widespread preservation technique used to extend the shelf life of food products. This process is performed in batch, semicontinuous, and continuous phases. HPP is based on the Le Chatelier principle, in which food items are exposed to high atmospheric pressures ranging from 300 to 700 Mpa, resulting in the decrease in fat globule size and the deactivation of enzymes such as plasmin. According to previous research, at pressures of more than 300 Mpa, the enzymes found in milk lose their action [47]. The HPP can be employed to preserve colostrum by optimizing conditions for reducing bacterial load and viral count with minimum alteration in bioactive components [36,47]. Gosch et al. [48] purposed a hurdle procedure for bovine colostrum that consists of a combination of HPP (400–500 Mpa, for 10 min) and microfiltration (1.4 and 0.8 m) that minimizes the microbial load to low or undetectable and has a less detrimental impact on immunoglobulin (IgG) than other types of thermal processing. More research is required to establish the effect of these combined processing procedures on the major and minor bioactive proteins present in bovine colostrum. Borad et al. [19] stated that spray drying reduced the total plate count (TPC; CFU mL^−1^) of BC samples by the value of 0.5.

The homogenization at different levels results in a significant change in the concentration of IgG, IgM, and IgA. The concentrations of IgG were 109.88 ± 3.04, 111.68 ± 2.15, and 110.63 ± 1.57 at homogenization levels of 10.34, 13.79, and 17.24 MPa, respectively [38]. Inabu et al. [49] measured glucagon-like peptide 2 (GLP-2) in bovine colostrum and mature milk by employing solid-phase competition immunoassay. GLP-2 is one of the gut-derived peptides cosecreted with GLP-1 from intestinal L cells in response to nutrient absorption. Freezing is the most frequent way to store and preserve raw colostrum, which is further associated with thawing [50]. Jones et al. [51] studied the effect of using microwave heating (325 W for 17 min and 650 W for 10 min) versus warm water (45 °C) to thaw the frozen colostrum samples, and based on results, the authors concluded that the microwave heating results in subtle coagulation with low protein content when compared to warm water. Furthermore, the authors also stated that there is no substantial difference in the composition of IgG and IgM in all the treatments. However, further research into the influence of repetition freezing and thawing on the concentrations of other main and minor elements of BC is required.

The nutritional and bioactive constituents of bovine colostrum are frequently substantial, and they are greatly influenced by processing conditions. Aside from altering the composition, these processing techniques promote the functional properties of BC-based products, reduce microbial load, and increase shelf life. Furthermore, these processing technologies are routinely performed in the manufacturing of a variety of BC-based products, such as IgG-concentrate powder, colostrum powder, and purified major and minor proteins, which are further extensively exploited in the manufacturing of a wide range of pharmaceuticals, cosmetics, and food and feed additives.

### Other Aspects That Might Influence the Composition of Bovine Colostrum

A number of different internal and external factors influence the composition of bovine colostrum, including cattle age (primiparous or multiparous), health status (udder diseases), herd factor, environmental (heat–cold stress), genetic makeup, feeding behavior and diet, parity, and dry period [1,8,52]. Xin et al. [53] evaluated the concentrations of odd-chain fatty acids (OCFAs) and branched-chain fatty acids (BCFAs) in BC and transition milk, as well as their stability, under various freezing (−20 °C for 30 days), heating (65 °C for 60 min), and heating/freezing combinations. During the study, samples from Holstein cows (*n* = 12) were collected at different intervals after parturition: colostrum (first day), transition (fifth day), and mature milk (ninth day). In colostrum, the amount of OCFAs + BCFAs was 134 mg/100 g, which was 24% lower in transition and 35% lower in mature milk. In all of the samples, C15:0 and C17:0 were the most prevalent fatty acids. Freezing and heating treatments had no significant effect on the stability of OCFAs + BCFAs. Similarly, O’Callaghan et al. [54] reported that the parturition or milking time, as well as lactation, have a considerable impact on the fatty acid (FA) profile concentration. In colostrum, for instance, the levels of PUFAs and saturated fatty acids were higher. The most affected component is conjugated linolenic acid (CLA), whose concentration was higher on the first lactation than on the third lactation. Furthermore, the concentration of FAs, C16:0, was shown to be greater in multiparous cows. In addition, the concentration of IgG in colostrum is adversely associated with the level of performance of bovine species, implying that high-yielding cows have a minimal percentage of IgG in their colostrum [55].

The fermentation process by lactic acid bacteria or probiotics, namely Lacticaseibacillus rhamnosus, L. acidophilus, Limosilactobacillus fermentum, Lacticaseibacillus paracasei, Enterococcus faecium, and Enterococcus faecium, may be a viable option for colostrum preservation [56]. Furthermore, Bartkiene et al. [18] evaluated the influence of fermentation with Lactobacillus paracasei LUHS244 and Lactobacillus plantarum LUHS135 together with dehydration and ultrasonication on the antimicrobial activity and immunoglobulins (IgG, IgA, and IgM) in BC products. From the findings, the authors suggest that IgA is most susceptible to fermentation, where the fermentation of BC with Lactobacillus plantarum results in minimal loss of IgG. The quality of bovine colostrum is determined by the lactic acid bacteria (LAB) strain used for fermentation. Another study conducted by Bartkiene et al. [57] indicates that the fermentation process, when combined with dehydration treatments and ultrasonication, allows for biological and moderate preservation of BC by lowering its microbial load, but it can also result in the formation of biogenic amines from proteins. Cummins et al. [58] reported that a temperature of ≤4 °C is ideal for storing colostrum samples while studying the impact of keeping colostrum in various conditions for varied storage times.

## 3. Recent Advancements in the Isolation and Identification of Novel BC Components

Researchers, technologists, and research and development organizations across the world are continuously working to develop novel technologies for the exhaustive understanding of the properties of colostrum constituents and to develop cost-effective and rapid methods for the isolation and identification of BC bioactive components.

Antibiotics are routinely used in veterinary practice for the treatment of various livestock ailments, and it is well known that prolonged exposure to antibiotics can cause a serious menace to wellbeing. Due to raised interest in animal-based products, the necessity of analytical methods for the identification of antibiotics in dairy-based products has been of serious concern. A simple and rapid method for simultaneous determination of antibiotics (*n* = 20) in BC-based tablets using ultra-high-performance liquid chromatography (UHPLC-MS)–tandem mass spectrometry and solid-phase extraction (SPE) was proposed by Zheng et al. [59]. Based on the experimental results, the authors reported that the use of UHPLC-MS together with hydrophilic–lipophilic balance (HLB) cartridge showed good extraction efficiency, low detection limit, and linearity as compared to the SPE method, which is onerous and required several pretreatments before eluting. Furthermore, the proposed technique is rapid, precise, and reliable in the simultaneous detection of common antibiotic residues of sulfisoxazole, clindamycin, roxithromycin, ofloxacin, sulfamoxole, ofloxacin, and so on in bovine colostrum-based tablets. An et al. [60] reported that near-infrared spectroscopy can be exploited for the rapid and precise qualitative detection of adulterated bovine colostrum.

Lee et al. [61] discovered the recovery of unique high-molecular-weight oligosaccharides composed of N-acetyl hexosamine in BC whey permeate. In their experiment, BC whey permeate was hydrolyzed with *Aspergillus oryzae* galactosidase for the removal of monosaccharides by membrane separation. From an industrial standpoint, this approach can be employed to transform BC whey permeate into novel oligosaccharides which further can be exploited as bio-therapeutic ingredients. The purification of colostrum immunoglobin (IgG) using salting-out precipitation technique was carried out by Skalka et al. [62] by using sodium sulfate and ammonium sulfate as a salt medium followed by cross-flow filtration (100 kDa; membrane pressure 0.15 and 0.08 Mpa at 25 °C); it resulted in the separation of up to 90% of the IgG fraction and a yield of up to 91%. Furthermore, the purified product can be used to make a variety of functional food and pharmaceutical items. Lactose hydrolysis and nanofiltration at pH 8.5 and 20 bar can be used to isolate high-purity bovine milk oligosaccharides from colostrum whey permeate or other milk sources, which can then be used to prepare infant formula [63]. Besides these, some of the advanced techniques employed in the identification and processing of bovine colostrum are presented in Table 2.

## 4. Bovine Colostrum as Modern Deliverable Nutraceutical Formulations

Increased consumer demand for foods that promote nutrition and health encourages food manufacturers and researchers to innovate safe and cost-effective food that can provide multiple benefits in a single entity [69]. Consequently, bovine colostrum (BC) and its derivatives have attracted researchers to use their biologically active constituents to make functional food and feed supplements [28]. BC is widely used as a food and feed supplement, and it is regarded as safe from a toxicological point of view [70].

### 4.1. Food Supplement

The toxicological and safety (in vitro, in vivo, and chromosomal aberration) evaluation of ultrafiltrate BC in accordance with international standards was studied by Thiel et al. [71]. In the study, Wistar rats were supplemented with different levels of ultrafiltered colostrum product at 1050, 2100, and 4200 mg/kg body weight/days for 90 days repeated. Obtained findings from the genetic toxicological study suggest that UF colostrum does not possess adverse effects in terms of mutagenicity, genotoxicity, clastogenicity, and mortality after 90 days of oral administration. Another study that supports the safety of bovine colostrum was undertaken by Davis et al. [72]. In their experiment, Lewis rats (*n* = 20) were supplemented with 3% and 5% BC powder and 10% skim milk powder as a control for 90 days. At the end of the study, no difference was observed in terms of food consumption; body weight; hematology; and blood chemistry, namely kidney and liver function, in both groups, except for reduced serum cholesterol concentration observed in rats supplemented with 10% colostrum.

Bovine colostrum is significantly rich in biologically active peptides, antioxidants, anti-inflammation agents, and growth-promoting factors that differ substantially from mature milk. The benefits of BC are well known in the health and disease of children and adults [73]. In view of these observations, BC is an emerging nutraceutical, and innovative therapeutic products are being developed for children’s formulas used for the growth and development of children as well as for treating GI tract diseases. Many researchers have shown that colostrum plays a critically significant role in the growth and maturity of the GI tract in infants. The nutrients in colostrum create a suitable environment, namely biochemical, physiological, morphological, functional, immunological, and antimicrobial, for the maturity of the gastrointestinal tract of neonatal piglets [74,75]. Newborn piglets are used as animal models for evaluating the antimicrobial properties, immune function, and maturation of the GI tract. Chae et al. [76] have reported that BC has strong anti-inflammatory and antibacterial activity in in vitro models. Lactoferrin present in BC can prevent gastric infections, necrotizing enterocolitis, and late-onset sepsis in newborn infants [77,78]. Hopefully, BC supplements will greatly contribute to curing different ailments in children and adults such as necrotizing enterocolitis, inflammatory bowel disease or Crohn’s disease, autoimmune disorders, upper respiratory tract infections, cardiovascular diseases, diabetes, and some cancer types [2,79,80].

Humans have historically consumed bovine colostrum, and a number of studies have indeed been conducted to investigate its possible benefits in human health and nutrition. A group of researchers conducted an animal trial to determine the effectiveness of added BC with human milk in the prevention of gut dysfunction and necrotizing enterocolitis in comparison to formula-based fortifiers. In his experiment, preterm pigs (*n* = 61) were divided into three groups and supplemented with two commercial milk formula-based fortifiers with donor human milk together with 80 g of BC powder for 5 days. Results from the study suggest that fortified BC with donor human milk reduces the risk of microbiota dysbiosis, intestinal dysfunction, and necrotizing enterocolitis [81]. Juhl et al. [25] exploited bovine colostrum in a randomized controlled pilot trial in which preterm infants (*n* = 40) were fed mother’s milk with BC powder and pasteurized human donor milk (DM). The study’s results indicate that supplementing BC with mother’s milk is tolerable, with progressively increasing enteral protein intake in infants, and increased plasma tyrosine level was also observed on the seventh day, without any clinically relevant adverse effects. Bovine colostrum can be foreseen as a promising alternative fortifier in infant formula that promotes intestinal health, nutrient absorption, and defense mechanisms. It is necessary to standardize the fortifier composition, and clinical trials should be undertaken precisely before fortifiers are fed to preterm infants. A study conducted by Y. Li et al. [82] suggests that BC powder was well accepted by newborns in a three-phase pilot trial, and more research is being conducted. Gao et al. [83] reported that bovine colostrum fortifier enhanced in vitro antimicrobial activity of human milk. Furthermore, the authors also concluded that a gently processed BC-fortifier could be a preferable fortifier for preterm infants when compared to a bovine milk fortifier.

Due to the prevalence of innumerable bioactive constituents in BC, including immunoglobins (IgG, IgA, and IgM), lactoferrin, growth factors, and many others, upgraded technology allowed researchers to formulate innovative foods, such as cheeses, tribiotics, probiotics, ice creams, candies, and yogurts, that provide a variety of health benefits beyond basic nutritive value. Reyes-Portillo et al. [84] synthesized bovine α-lactalbumin made lethal to tumor cells (BAMLET) complex from oleic acid and bovine colostrum (second and third milkings) for the development of functional cheese spread. The spread prepared from the second milking of bovine colostrum showed a yield of about 40% with high protein content, i.e., 13.56%, as compared to the spread made from the third milking. However, both spreads showed good inhibitory activity, antihypertensive capacity, low adhesiveness, and uniform soft texture with satisfactory sensory acceptability.

Ice cream is another successful food product manufactured from bovine colostrum. For the preparation of ice cream, BC was added at different concentrations of 0.400, 0.800, 1.200, and 1.600 kg with milk, butter, SMP, sugar, stabilizer, and flavoring agent. Ice cream prepared with 1.600 kg of colostrum with 2.400 kg milk and other ingredients exhibited 5.98% carbohydrates, 5.37% protein, 19.55% radical scavenging activity, and less overrun (around 19%). The authors also suggest that approximately 30–40% colostrum can be used in the production of ice creams that exhibit good antioxidant properties and other nutritional parameters [85].

The popularity of functional confectionery products is continuously rising due to their high acceptability and extended shelf life, which makes candies a potential supplement for both adults and children. Bartkiene et al. [86] proposed a unique formulation for the production of gummy candies by exploiting the bovine colostrum (2 h after calving), oil-in-water extracts of essential oils (clove, grapefruit, thyme), and probiotic strains (*Lactobacillus paracasei* LUHS244 and *Lactobacillus plantarum* LUHS135). Gummies prepared with 3% fermented bovine colostrum, with 0.2% essential oil from thyme, with 0.2% grapefruit or mandarin exhibit considerable texture, good antimicrobial activity, and sensory acceptance. These types of formulations could well be preferred as nutraceuticals by consumers, and they may play a key role in the food sector by conferring nutrition and health advantages in a single entity.

Cotârleţ et al. [87] transformed BC into a tribiotic product through two-step biotransformation, i.e., enzymatic conversion and fermentation. For the production of tribiotic products, BC was initially enzymatically transformed for 48 h with yeast strain *Candida lipolytica* followed by lactic acid fermentation (LAB) for another 48 h with kefir grains. The prepared product can be used as a functional ingredient of milk-derived nutraceutical formulations that display good antioxidant potential. A new variant of yogurt fortified with bovine colostrum (at the different levels of 5%, 10%, and 15%) with date syrup (5%) was proposed by Abdel-Ghany and Zaki [88]. Prepared yogurt with the incorporation of BC at the levels of 10% and 15% showed high viscosity. Moreover, all the BC-based samples had an adequate nutritional composition and sensory acceptability. The ultrafiltration could be utilized to produce an IgG-rich retentate, which can then be used to produce IgG-enriched cheese or “Domiati” [89].

Several products based on bovine colostrum and its derivatives are available on the market, including Igazym lozenges comprising colostrum powder and lysozyme (Pedersen Biotech, Vejle Denmark); Naco IgG Plus colostrum skim milk (Smart Naco, SDN, Malaysia); Extra Edge colostrum powder and Emma colostrum-based cream (Immuno-Dynamics Inc. Fennimore, WI); ColoPlus (ColoPlus^AB^, Malmo, Sweden); Lactobin spray-dried Ig-enriched powder (Biotest Pharma, Dreieich, Germany) [90]; Gastrogard-R hyperimmune colostrum concentrate (antirotavirus), colostrum concentrate (Intact); sterile-filtered colostrum-based product (Viable Bioproducts, Ltd., Turku, Finland); Immunova, the world’s first drinkable colostrum-based product (Novatreat Ltd., Turku, Finland) [91]; spray-dried colostrum powder (PuraLife, Harrisburg, PA, USA); capsules (Jarrow Formulas, Robertson Blvd, Los Angeles, CA, USA); colostrum capsules; and powder (Sovereign, Cottonwood, AZ, USA) [92]. Numerous interesting commercialized products based on bovine colostrum and its derivatives and their prospective advantages were eloquently elucidated by [90,91,92].

With the advanced scientific understanding, worldwide standards, and sophisticated equipment, researchers now can synthesize colostrum-constituent-based nanoparticles and nanoemulsions, which may then be employed as vehicles or carriers of bioactive components. However, there are several challenges and opportunities for the therapeutic applications that need to be addressed and warrant further studies. For instance, understanding the biological roles of different BC ingredients is a major challenge for nutritionists and dieticians, basic researchers, and physicians. Furthermore, well-designed, placebo-controlled, and randomized clinical trials are needed to determine the long-term safety, effectiveness, and optimal doses of BC supplements. Some other aspects of BC nutraceuticals include the standardization of products originating from different breeds of cows, buffaloes, and other animal species. In addition, good manufacturing practices and standardized techniques are required for making BC formulations and preventing possible adulteration of BC supplements with synthetic drugs and microbial contaminants, just to name a few. More basic research is needed to understand the mechanism of action of different components of BC for immune-boosting properties, treating inflammatory bowel diseases and gastrointestinal disorders, antidiabetic and anticancer effects, and curing upper respiratory tract infections.

### 4.2. Feed Supplement

Over the past few years, researchers and food manufacturers have also shown keen interest in using bovine colostrum and its derivatives as a feed additive. Tay et al. [93] conducted a study on cynomolgus macaques (*Macaca fascicularis*) to determine the supplementation effect of bovine colostrum together with human milk formula on bone mineral density (BMD). Cynomolgus macaques (*n* = 24) of age 5–6 years were divided into four groups and supplemented with (a) breastfeeding, (b) formula-feed, (c) formula and IGF1, and (d) formula with bovine colostrum. The results from the study indicate that group (d) resulted in a significant increase in BMD as compared to other tested groups. This study can be used as a model for developing an infant’s food by standardizing the composition as per neonate body requirements.

The supplementation effect of lyophilized BC whey powder as a protein feed source for farm fish (*Piaractus mesopotamicus*) was demonstrated by da Cruz et al. [26]. During the experiment, farm fish (juvenile pacu) in controlled conditions were administered with lyophilized BC at different levels, i.e., 0%, 10%, and 20%, for 60 days with pelleted experimental diets. After 30 or 60 days, fish were analyzed for morphometric parameters including muscle layer thickness and goblet cell distribution in the intestinal epithelium. From the experimental results, the authors reported that the 20% supplementation of BC for 60 days results in the thickening of muscle layers of the intestinal epithelium and also elevates nutrient absorption and concentration of goblet cells. Lyophilized BC whey powder can be used as an alternative feed source for neotropical fish as it offers a variety of bioactive components and excellent protein sources.

Bovine colostrum can be exploited as a feed additive in poultry, particularly in broiler chickens, to maximize protein quality and body weight and to decrease feed cost. A supporting study that indicates that BC can be used be as an alternative to poultry feed to enchase the bodyweight of broiler chickens was conducted by Afzal et al. [27]. Broiler chickens (*n* = 192) of 3 days were supplemented with basal diet as control and BC at different levels (1%, 1.5%, and 2%/kg) and assessed daily for body weight and mortality. Experimental data disclose that broiler chickens supplemented with 2% BC/kg showed the highest body weight among all the treatments, with the lowest mortality rate.

Besides the aforementioned, researchers across the globe continuously focus on formulating feed with bioactive constituents for Aves. A recent study examining the supplementation effect of BC powder on the performance, serum lipid peroxidation level, and carcass yield in Japanese quail was conducted by Akdemir et al. [28]. For the experiment, Japanese quail (*n* = 90) were divided into three subgroups and supplemented with a basal diet, a basal diet with 2.5% BC powder, and a basal diet with 5% BC powder for 42 days. Feeding a basal diet with 5% BC powder significantly enhanced the overall performance concerning body weight, gained weight, and carcass weight. However, a reduced serum lipid peroxidation level was also observed in the group fed with 5% BC powder together with basal diet. For a long time, products based on bovine colostrum whey have been marketed as colostrum substitutes or colostrum supplements for calves [94]. When colostrum has a low immunoglobulin concentration, colostrum substitutes provide calves with an extra dose of immunoglobulins, whereas colostrum supplements can be administered in place of maternal colostrum. More research utilizing bovine colostrum or its derivatives in the formulation of feed supplements for various domesticated or farm animals that minimize the health-economic load of owners is likely to be conducted in the future years.

## 5. Current Evidence of Therapeutic Use of BC

For hundreds of years, bovine colostrum has been exploited as a traditional or alternative medicine for a wide range of medical conditions. Some of the recent studies that display the medicinal actions of bovine colostrum and its derivatives are summarized below.

Intestinal permeability or hyperpermeability is a recently observed medical disorder in which the tight connections in the gut epithelial wall lose their integrity, allowing material from the lumen to leak into the bloodstream or other organs [95]. Hałasa et al. [96] conducted a placebo-controlled, double-blind trial on healthy individuals to determine the effectiveness of BC supplementation in responses to intestinal permeability, conducting a further assessment with the mannitol/lactulose differential sugar absorption test. For the study, the colostrum samples were collected at different lengths of time after parturition (2, 24, and 72 h). The healthy volunteers (*n* = 31) were divided into four subgroups and supplemented with 500 mg of colostrum powder twice per day for 20 days, i.e., group 1 (colostrum collected at 2 h), group 2 (colostrum at 24 h), group 3 (colostrum at 72 h), and group 4 (placebo). Individuals are assessed for mannitol/lactulose differential sugar absorption test from baseline to end of the study. From the outcome of the study, the authors suggested that the supplementation of colostrum collected within 2 h (group 1) is more effective in reducing intestinal permeability. Moreover, the quality of colostrum-based food products can be determined by their bioactivity with a mannitol/lactulose test.

Another study that supports the above-mentioned study and the positive effect of BC supplementation on intestinal permeability was conducted by Eslamian et al. [97] on critically sick patients (*n* = 70). Patients (*n* = 32) were supplemented with BC powder at a rate of 20 g/day, and *n* = 30 were supplemented with isocaloric maltodextrin as a placebo. Patients were observed and their samples were analyzed from baseline to end of the study. Obtained results indicate that the patients supplemented with BC were observed to have less incidence of diarrhea and reduced levels of zonulin and plasma endotoxin. Furthermore, supplementation of BC may have favorable benefits on intestinal permeability, although more research is needed to determine the specific mechanism. Similarly, Barakat et al. [98] demonstrated the supplementation effect of BC on children in the treatment of acute diarrhea. Children (*n* = 160) of age 2 months to 6 years who suffered from acute diarrhea were supplemented with BC (6 h) comprising 350 mg IgG, 35.3 mg IgA, and 25.3 mg IgM mixed with 50 mL water and placebo (identical to BC) for one week. Results obtained after 48 h revealed that children supplemented with BC had a lower frequency of diarrhea and vomiting. BC supplementation was effective in the treatment of both bacterial (*E. coli*) and viral (rotavirus) related diarrhea complications. Florén et al. [99] conducted an open-labeled observational study on *n* = 30 subjects from Nigeria, in which the subjects were supplemented with 50 g of a bovine colostrum-based product “ColoPlus-colostrum powder—32% (3–4 g of IgG/50 g), maltodextrin, rice and banana flakes” twice a day. The outcome of this study suggests that the consumption of these BC-based products reduced HIV-associated diarrhea and the daily number of bowel evacuations and increased CD4^+^ cell albumin and hemoglobulin concentrations. ColoPlus might be an effective alternative or supplemental therapy for HIV-related diarrhea which further needs control studies.

Human respiratory syncytial virus (RSV) is a common, contagious virus that causes respiratory infections, namely bronchiolitis, common cold, and serious illness for infants and elderly persons. To study the preventive action of isolated IgG from bovine colostrum in response to RSV, an animal trial was carried out by Nederend et al. [100]. BC was collected within 5 days after parturition, and IgG was isolated from whey by using affinity purification and evaluated for a binding property with culture HEp-2 cells infected with two strains of RSV in a mouse model (in vitro). The obtained finding reveals that BC-isolated IgG has the potential to neutralize RSV. The authors also suggested that purified IgG be added to infant formula, which could have protective effects in contrast to raw milk or colostrum and also be microbiologically safe. The BC antibodies showed positive results (in vitro) in the reduction in infection from bovine leukemia virus (BLV) in neonate calves [101], where BC-derived dietary exosomes encourage antiosteoporosis activity in vivo and in vitro [69]. The IgG from bovines assists in the modulation of intestinal cells and increases the adherence of bifidobacteria [102].

A case study presented by Alsayed et al. [29] showed the effectiveness of BC in preventing upper respiratory tract infection (URTI) and nasal swab microbiome caused by respiratory viruses in an adult. This study was conducted on a 27-year-old patient who was a nonsmoker and nonalcoholic and took a healthy diet with supplementation in three periods: period a, 1 g of bovine colostrum (two capsules, each 500 mg) for 4 weeks; period b, 2 weeks after the end of the period a, 1 g BC at dawn for 3 days; period c, after period b, supplemented with 1 g BC at dawn 2 times/week for 4 weeks. This supplementation of BC is effective in the treatment of upper respiratory tract infection and nasal swab microbiome. Hence authors recommend this treatment for individuals suffering from URTI. Similarly, Oloroso-Chavez et al. [103] also reported that supplementations of BC (1000 mg for 3 months) improve lung function and nasal congestion in a monosensitized subgroup (individuals suffering from respiratory allergies).

The effectiveness and safety of a purified BC-based medical device, i.e., Monurelle Biogel, a vaginal gel used in the treatment/prevention of vulvovaginal atrophy and other urinary symptoms in postmenopausal women, were studied by Schiavi et al. [104]. For the study, postmenopausal women (*n* = 172) with vulvovaginal atrophy were advised to apply gel (5 mL/each) for 12 weeks either after intimate cleaning or before sexual intercourse. The outcome of the study revealed that this BC-based medical device has potential in the treatment of vulvovaginal atrophy; it may improve sexual life, assist in the other urinary symptoms, and overall enhance the quality of life.

Lately, isolated LF from BC has indeed been extensively used as a functional component in the manufacture of infant formula, cosmetics, and nutritional supplements [105]. The usage of lactoferrin is Generally Recognized as Safe (GRAS) approved by the US Food and Drug Administration (FDA), and the maximum limit of LF in food products was also established by European Commission (EC) No. 258/97 [69]. According to Lepanto et al. [106], milk-derived LF has the potential to chelate two ferric ions per molecule and can be seen as a natural anti-inflammatory substance that has the potential to modulate ferroportin and hepcidin synthesis by the downregulation of interleukin-6. Supporting the study of Lepanto et al. [106], the potential of bovine LF in the treatment of Fe-deficiency anemia was also reported by Taruni et al. [107]. El-Khawaga and Abdelmaksoud [108] stated that the LF can be an excellent alternative in the treatment of Fe-deficiency in school-going children. The antiviral property of LF can extendable in the treatment of Zika and the Chikungunya virus [109]. LF showed antiviral activity (in vitro) against SARS-CoV; based on these functional and iron chelator properties, authors concluded that LF might show virucidal action against SARS-CoV-2 [110] and warrants detailed study. The supplementation of 32 mg bovine liposomal lactoferrin/10 mL with 12 mg of vitamin C—four to six doses/day for 10 days—was found to be effective in the prevention of COVID-19 infection [111].

Tanideh et al. [112] examined the efficacy of BC (0.5–1 mL) in combination with honey (0.5–1 mL) in the healing of cutaneous wounds in an animal trial. Sprague Dawley rats (*n* = 80) were divided into four groups: group 1 (control; no treatment), group 2 (treated with honey), group 3 (treated with BC), and group 4 (BC and honey). Wound healing was monitored on camera on days 3, 7, 14, and 21. Based on the obtained findings, the authors reported that the combination of honey and BC enhances wound healing. Moreover, the dressing with BC and honey on wounds reduces scars and exudates, provides pain relief, protects from infection, and stimulates the growth of the granulation tissue.

Necrotizing enterocolitis is a severe gastrointestinal disease that often affects preterm newborns’ intestines. The ability of oral BC supplementation to preterm neonates to reduce the risk of necrotizing enterocolitis (NEC) and late-onset sepsis (LOS) was studied by Awad et al. [113] in a placebo, double-blinded, and randomized trial. Preterm neonates (n = 80) of age < 34 weeks were divided into two groups supplemented with BC concentrate and placebo for 2 weeks. The early hypocaloric feeding with BC concentrate reduces the risk of morality, NEC, LOS, and feeding tolerance in preterm neonates. The authors also recommended the BC usage as gut priming. The ability of BC supplementation in the reduction in linear growth faltering and environmental enteric dysfunction (EED) in (n = 267) Malawian children of age 9–10 months was examined by Bierut et al. [114] in a randomized, controlled clinical trial. Children (*n* = 267) of age 9 months were supplemented with either BC (5.7 g) with whole egg powder (4.3 g) or placebo (unfortified soy/corn blend; 15 g) twice a day for 12 weeks. The supplementations of BC/whole egg powder to complementary feeding result in the reduction in linear growth faltering.

The lyophilized bovine colostrum (collected in 1 h after parturition with 29.6% IgG and 71.2% protein, defatted) at the level ranging from 0.05% to 0.5% for 24–48 h was found to be beneficial in the treatment of skin disease or keratinocytes in UVR-exposed subjects or cutaneous dryness in old age persons [115]. Fajardo-Espinoza et al. [116] reported that the BC whey protein hydrolysates (WPHs) obtained from in vitro digestion with pancreatin and pepsin have the immunostimulatory potential in the pretreatment of chronic disease, including cancer. The supplementation of BC played a protecting role in the regulation of inflammatory responses [117] and hepatic fibrosis in different liver diseases [118].

To see the effect of these preventive actions of BC on 2,4,6-trinitrobenzene sulfonic acid induced colitis in mice, a study was conducted by Filipescu et al. [119]. Mice (*n* = 24) were divided into groups, group a were fed with a suspension of BC (100 mg of colostrum in 0.6 saline solution) and the same volume of only saline as control. Experimental data results revealed that the BC supplement having bioactive peptide is effective in the pretreatment of or reduction in intestinal damage or inflammatory bowel disease and other clinical signs of colitis. Additional studies are needed to better understand the mechanism behind the immunomodulatory action. The oral administration of BC IgG-enriched whey fractions (IgG25+) has the potential to protect against food-borne infections (*Salmonella enterica* serovar Enteritidis, Enterohaemorrhagic *Escherichia coli* O1 57:H7, *Mycobacterium avium*) and was also found to be partially effective against respiratory tract infection in mice [120]. Sanctuary et al. [30] reported that the supplementation of bovine colostrum oligosaccharides with *Bifidobacterium longum* subspecies *Infanti* 0.3 g twice/day for 12 weeks to children of age 2–11 years suffering from gastrointestinal symptoms and autism disorders results in improvement of gastrointestinal (GI) symptoms and is well-tolerated with a mild gassiness as a common side effect.

Numerous studies have been conducted across the world in recent years to assess the potential of BC and its derivatives; however, there have been a few studies that show that bovine colostrum alone is insufficient to treat all illnesses. For instance, Sadeghirad et al. [121] did not observe any significant effect of colostrum from human and bovine colostrum on necrotizing enterocolitis. Another study conducted by McKenna et al. [122] revealed that supplementation of BC does not improve salivary lysozyme and LF at rest and in postexercise conditions. Furthermore, well-designed, placebo-controlled, and randomized clinical trials are needed to determine the long-term safety, effectiveness, and optimal doses of BC supplements.

## 6. Concluding Remarks

During the last several years, the bovine colostrum and its derivatives have emerged as a topic of scientific study that attracts academics, researchers, food manufacturers, and healthcare practitioners. Apart from internal and external factors, the composition of bovine colostrum and its derivatives changes markedly with the processing conditions and techniques such as heating, freezing, homogenization, and other chromatographic methods. In addition, these approaches also assist in extending the shelf life of BC-based products by reducing the microbial load with minimum loss of bioactive components. Cutting-edge technologies and sophisticated instrumentation have created opportunities for researchers and food manufacturers to utilize BC components in the formulation of safe, nontoxic, cost-effective, nutritious food and feedstuff that simultaneously offers a wide range of therapeutic and nutraceutical benefits. The split trehalase immunoglobulin G assay (STIGA) and refractometer could be foreseen as promising tools for the quantitative estimation of IgG at the laboratory and farm levels. Bovine colostrum and its derivatives, namely hydrolysate, isolates, and purified constituents, are extensively exploited in the formulation of nanoemulsions, tribiotics, cosmetics, yogurts, and other products that exhibit substantial functional properties and considerable sensory acceptability. The potential use of bovine colostrum for preterm neonates is being investigated. Fermented bovine colostrum is a good source of bioactive peptides with increased biological activity, and lactic acid bacteria fermentation combined with other preservation techniques such as ultrasonication might be a feasible choice for mild colostrum preservation. More studies are warranted to determine the effect of these treatments on the bioactive constituents of BC. Aside from these dietary supplements, BC components are progressively being used in the production of livestock feed, which is effective in delivering enough protein for muscle development and overall body development without causing mortality. Furthermore, the biomedical and pharmaceutical companies are looking forward to developing safe, durable, and efficacious healthcare medications or supplements by exploiting the bioactive components of bovine colostrum. The prophylactic action of hyperimmune components against bacterial and viral illnesses is well documented in folk and modern drug delivery systems. Evidence from clinical trials also suggests that the supplementation of BC and its derivatives has potential in the pretreatment of leaky gut syndrome, necrotizing enterocolitis, IBD, respiratory tract infections, acute diarrhea, and other immune-related disorders. Further studies are needed to understand the mechanisms by which colostrum bioactive constituents act as antimicrobial, anti-inflammatory, anticancer, antiobesity, and antidiabetic agents as well as a cure for IBD. However, some people may experience gastrointestinal problems such as flatulence and nausea. It has been suggested that the hyperimmune colostrum constituents such as Igs and lactoferrin may be used as an alternative therapy to construct antibodies against COVID-19 infection.

## Figures and Tables

**Table 1:** Difference in immunoglobulin concentration between human and bovine colostrum.

Immunoglobins			
Bovine (mg/mL)	Milk	Colostrum	Serum
IgG_1_	0.59	20–200	14.0
IgG_2_	0.12	12.0	11.0
IgM	0.05	4.20	3.1
IgA	0.14	3.90	0.4
**Human (mg/mL)**			
Total IgG	0.04	0.43	12.1
IgM	0.10	1.59	0.9
IgA	1.00	17.35	2.5

Data adapted from [14,15,17].

**Table 2:** Modern techniques employed in the isolation and identification of BC components.

Technique/Component	Study Aim	Subject/Sample	Key Findings	Reference
Isobaric tags for relative and absolute quantitation (iTRAQ)-coupled LC-MS	Proteomic analysis of whey protein in BC, mature milk, and human milk.	Colostrum (0–5 days) collected from Chinese Holsten (*n* = 30), mature milk (15–6 months), and human milk (*n* = 60).	iTRAQ-coupled LC-MS is the most advanced, reliable, and precise technique in the proteomic quantification of milk from different origins and lactation.	[64]
Laser-perturbation 2-D correlation Raman spectroscopy	Exploration of laser perturbation 2-D correlation Raman spectroscopy method for the reliable and rapid assessment of the quality of BC-based products.	Colostrum samples in milligrams were loaded in porous chips, followed by the Raman spectral analysis. The excitation wavelength was 785 nm and laser power was 450 nm.	The intended approach is simple, rapid (<5 min), and inexpensive.$$$The correlation coefficient analysis assists in improving the difference in experimental samples and improved spectral resolution.$$$In the future, this approach might be utilized to effectively differentiate milk powder in BC-based products.	[65]
UHPLC-QTOF-MS	Characterize and compare the lipids in mature milk and BC based on ultra-high-performance liquid chromatography–quadrupole time of flight mass spectroscopy (UHPLC-QTOF-MS) lipidomics.	Mature milk and BC.	A total of 335 lipids belonging to 13 subclasses were identified in both mature milk and BC.$$$*n* = 63 lipids were significantly different in mature milk and BC. Out of 63 significantly different lipids, SDLs (*n* = 21 SDLs) were found to be higher in BC; the rest (*n* = 42 SDLs) were found to be in an elevated concentration in mature milk.	[3]
Split trehalase immunoglobulin G assay (STIGA)	Develop a novel method for the estimation of IgG in BC and serum at the farm level.	BC (*n* = 60), calf serum (*n* = 83), and purified bovine IgG (12.8 mg/mL) used as standard. This STIGA is based on the enzymatic action of trehalase (TreA), which converts trehalose into glucose which is further detected by glucometer.	STIGA is a single step assay that requires less time as compared to other methods which directly measure IgG and could be a promising method to use for the detection of IgG at the farm level.	[66]
Dye affinity chromatography	Purification of lactoperoxidase (LPO) from whey by employing dye affinity chromatography.	Triazine dye (*n* = 18) was immobilized on Sepharose 6B, followed by the screening of their activity as possible ligands.	Dye–Sepharose (*n* = 5) matrix showed more than 90% adsorption of LPO without any pretreatment. The Reactive Red-4 Sepharose matrix can be used in the one-step purification of LPO from bovine whey.$$$The dye affinity chromatography has potential in the purification and recovery of LPO from bovine whey by using different chromatographic support.	[67]
Mixed-mode chromatography	Develop an efficient, cost-effective procedure to isolate pure IgG from colostrum whey with minimal activity loss.	Two modes were used to separate the IgG from colostrum whey. Capto multimodal chromatography material (MNC) and MEP HyperCel matrix were used to capture IgG.	The authors isolated pure IgG (130–150 g) from 3 L of whey in five hours. This mixed-mode chromatography results in the purity of 96.1% IgG. This technique can be used in the future for the pure, stable, and active isolation of IgG from bovine milk and colostrum.	[68]
Transmission infrared (IR) spectroscopy and Brix refractometer	Determine the effectiveness of transmission infrared spectroscopy (IR) and Brix refractometer (optical and digital) in the estimation of colostrum IgG concentration.	Colostrum samples of Holstein cow (*n* = 258). The concentration of IgG was determined in 255 samples using infrared spectroscopy (IR) and radial immunodiffusion assay (RID). Colostrum samples (*n* = 240) were analyzed by using a refractometer (optical and digital).	Transmission infrared (IR) spectroscopy is an accurate and rapid method to determine the colostrum quality, i.e., IgG, in lab-based testing where the Brix refractometer (digital and digital) is less effective. Moreover, the study suggests that transmission infrared spectroscopy is effective at the lab scale, while the Brix (digital and digital) can be used to determine the colostrum quality at the farm level.	[21]
Exosomal microRNAs	Identify and compare the exosomal $$$microRNAs in both milk and colostrum.	Dogu Anadolu Kirmizisi and Holstein cows,	A total of 795 miRNAs were expressed and identified differently, out of which 545 were identified as miRNAs, of which 250 were identified as novel miRNAs. These miRNAs regulate milk protein and fat metabolism.	[22]
Neutral and acidic oligosaccharides	Isolation of neutral and acidic oligosaccharides from BC and goat milk for infant formula.	20 mL of BC (0–3 days) after parturition, human milk (*n* = 20) and goat milk were used for the isolation of oligosaccharides through the modified charcoal column method.	The oligosaccharides from goat milk have more potential in terms of adherence inhibition and safety to be utilized in infant formula.	[23]
N-glycoproteomes	Characterize and compare whey N-glycoproteomes from BC, HC, and mature milk.	A bioinformatics analysis using liquid chromatography (LC)–tandem mass spectrometry. Colostrum and mature milk were collected from Holstein cows (*n* = 60) and HC (*n* = 60). Further collected samples were divided into 3 pooled groups (*n* = 20).	N-Glycoproteomes were different in BC, HC, and mature milk. N-Glycoproteomes (68, 58, and 98) were identified in all milk samples. The composition of N-glycoproteomes is significantly changed with lactation.	[20]

## Data Availability

Not applicable.
